# Estimated diameter increase from a 4S to a 6S hamstring graft configuration – A cadaveric study

**DOI:** 10.1051/sicotj/2023033

**Published:** 2023-11-30

**Authors:** Yoan Bourgeault-Gagnon, Alexandre Keith Leang, Sonia Bédard, Karina Lebel, Frédéric Balg, François Vézina

**Affiliations:** 1 Sherbrooke University 3001 12th Avenue North Sherbooke QC J1H 5H4 Canada; 2 Centre Hospitalier Hôtel-Dieu-de-Sorel 400 Av. de l’Hôtel-Dieu Sorel-Tracy QC J3P 1N4 Canada; 3 Center for Research at the CHUS (CIUSSS de l’Estrie CHUS) 3001 12th Avenue North Sherbooke QC J1H 5H4 Canada; 4 Research center on aging (CIUSSS de l’Estrie CHUS), 1036, rue Belvédère Sud Sherbooke QC J1H 4C4 Canada; 5 Department of Electrical Engineering and Computer Engineering, Université de Sherbrooke 2500, boulevard de l’Université, bureau C1-3050 Sherbrooke QC J1K 2R1 Canada

**Keywords:** ACL reconstruction, Anterior cruciate ligament, Hamstring graft, Cadaveric study

## Abstract

*Purpose*: Graft diameter in anterior cruciate ligament reconstructions has been shown to influence the risk of failure. It is therefore important to be able to adjust the graft configuration to modify the diameter. To measure the impact of a 6-strand (6S) hamstring autograft configuration on graft diameter compared to the standard 4-strand (4S) configuration. *Methods*: Cadaveric study on 33 knees, using the usual hamstring graft harvesting technique. Semitendinosus and gracilis tendons were harvested and their length, width, and diameter were measured in 4S and 6S configurations separately by three evaluators. *Results*: 6S configuration leads to a median increase of 1.5 (range: 0.0–2.0) mm in diameter compared to 4S (*p* < 0.001). A graft diameter of more than 8 mm is attained in less than a third of 4S grafts within this population in comparison to 84% when the 6S configuration is used. *Discussion*: The 6S hamstring graft configuration increases the graft diameter by a median of 1.5 millimeters compared to the traditional 4S configuration. It can reliably be used to obtain an 8.5 mm graft diameter or more in cases where the semitendinosus measures at least 270.5 mm and the 4S configuration has a diameter of 7.5 mm or 8 mm. This information helps to better delineate the impact of a 6S configuration in a pre-operative or intra-operative setting to optimize the decisional process and surgical flow and to easily adapt the graft diameter.

*Level of evidence*: V (cadaveric study)

## Abbreviations

ACL: Anterior cruciate ligament
4S: 4 Strand hamstring graft
5S: 5 Strand hamstring graft
6S: 6 Strand hamstring graft
BPTB: Bone-patellar tendon-bone graft

## Introduction

The failure rate of anterior cruciate ligament (ACL) reconstructions remains high, between 0.7% and 20% [1]. The main factors related to an increased risk of revision can be grouped into biological, traumatic, or technical causes and include, among others, patient age, compliance with postoperative protocol, type of sport, tunnel placement, type of graft, graft diameter, and absence of associated lateral extra-articular reconstruction [1–6].

Historically, patellar tendon grafts (BPTB) were considered the gold standard, but hamstring grafts are now the most popular option internationally [7]. Their primary advantages are smaller incisions, a lower risk of anterior knee pain, and a high load-to-failure. Conversely, their main potential drawbacks are residual hamstring weakness, risk of saphenous nerve injury, difficulty in predicting graft size, and a potentially longer maturation time [8, 9]. This last factor is the one this project is based on since being able to adapt intra-operatively is crucial. It is important to consider the length and size of the harvested semitendinosus and gracilis tendons in order to obtain a graft of adequate diameter, as this influences its biomechanical properties [10, 11]. Graft diameter larger than 8 mm seems to be associated with a lower risk of failure and better functional results [2–4], as shown in the meta-analysis done by Conte et al. who found a relative risk of failure 6.8 times greater for grafts equal or less than 8 mm in diameter [3]. In addition, Spragg et al. demonstrated a reduction in the relative risk of failure of 15% per 0.5 mm increase in graft diameter [12, 13].

Classically, a 4-strand configuration (4S) has been used for every case, but a substantial proportion of grafts would not reach a diameter above 8 mm. Techniques have been subsequently described to increase graft size when appropriate. Depending on graft length, size, and specific patient’s characteristics, surgeons can decide whether to use a hybrid allograft/autograft [14], a partial width quadriceps tendon augmentation [15, 16] or, most commonly, modify the graft configuration to obtain a 5-strand (5S) or 6-strand (6S) hamstring autograft configuration [17].

To optimize graft diameter while not significantly affecting the surgical flow, multiple authors have established that the cross-sectional area of the hamstring tendons on a pre-operative magnetic resonance imaging (MRI) is significantly correlated with the intra-operative 4S graft size and can be used to refine surgical planning [3, 18]. Similar correlations have been shown for pre-operative anthropometric parameters like height, age, thigh diameter and length, and gender [3, 19, 20]. We propose that an adjunctive pre-operative and intra-operative predictive tool, designed to estimate the potential increase in diameter from changing a 4S to a 6S configuration, could be useful. This tool would either confirm the planned graft choice or allow for last-minute adjustments. Specifically, if pre-operative MRI measurements of the hamstring or intra-operative measurements of a 4S graft indicate an insufficient diameter, the calculated 4S-to-6S increment value could inform the decision on whether to proceed with the conversion or opt for an alternative augmentation method.

The objective of this descriptive cadaveric study was to measure the impact that a 6S configuration has on graft diameter compared to a 4S configuration. The hypothesis was that this technique reliably increases the diameter of the graft by more than 1 mm. The secondary objective was to establish the minimal semitendinosus length and 4S diameter necessary to reliably obtain 6S grafts with a diameter larger than 8 mm, i.e. 8.5 mm or more, and a length of 90 mm or more, considered as a safe minimum length for proper tibial fixation. Finally, intra-rater and inter-rater reliability were evaluated on diameter measures of 4S grafts.

## Methods

The protocol of this descriptive cadaveric study was approved by the local ethical committee. Between January and March 2019, 17 specimens were prepared according to the usual technique for fresh corpse preservation, without embalming. Thirty-four cadaveric knees were dissected by an orthopedic surgery senior resident. Inclusion criteria consisted of every cadaver that had previously given written consent to donate their body to science. Exclusion criteria included any previous surgery on the hamstring tendons or damaged tendons rendering them unusable for manipulation. No power analysis was performed; every specimen available during the data collection period was used, which was of the same magnitude as both previous studies of the subject (*n* = 23 [21] and *n* = 61 [22]).

**Figure 1:**
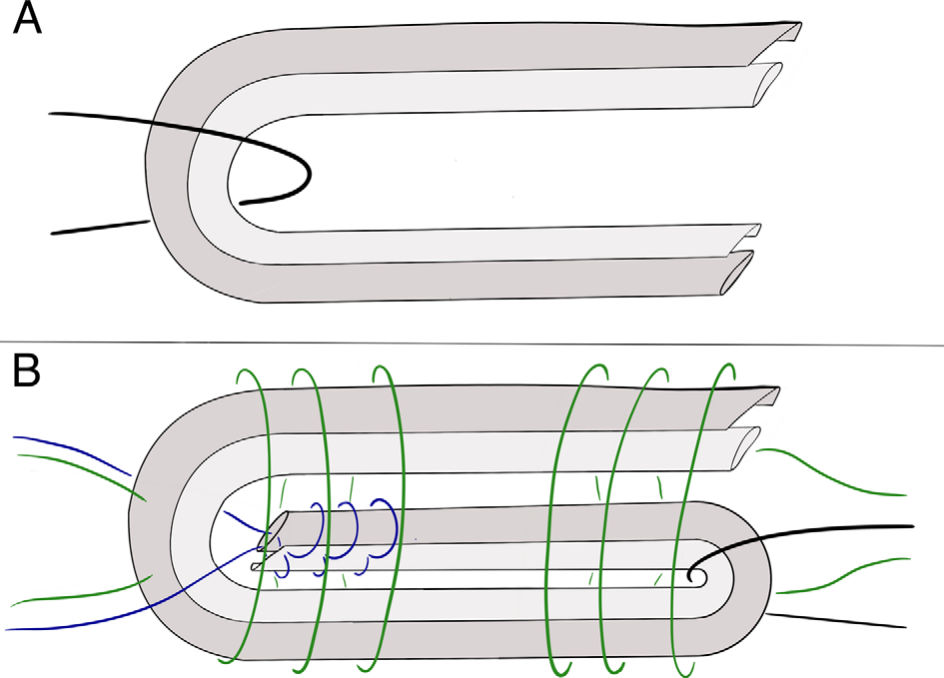
Illustration of semi-tendinosis (dark grey) and gracilis (light grey) tendons in 4S (A) and 6S (B) graft configurations used for diameter measurements. A: 4S configuration representing the initial diameter measurement before whipstitching. Both tendons are folded over a PDS suture before diameter measurement. B: 6S configuration representing the final graft diameter measurement. 4S graft is unfolded and both tendons are sutured together with a resorbable polyfilament suture (blue). The graft is then folded in 3 equal parts over a resorbable poly filament suture (black). Both ends are whipstitched and tabularized with a resorbable poly filament 2-0 suture (green) before diameter measurement.

Each tendon was harvested by the same senior resident using the usual semitendinosus and gracilis harvesting technique, as described thoroughly in a previous publication [15]. A *tendon harvester for cruciate ligament reconstruction* (Conmed Linvatec, Aurora, Ohio) was used through an oblique anteromedial incision to harvest both tendons after freeing them from their adherences and keep as much length as possible by dissecting the insertion subperiosteally. Each pair of tendons was then stored in a freezer until all the specimens were ready to be used. Measurements were completed after two measuring sessions. The frozen tendons were gradually thawed at room temperature and kept moist in a surgical sponge dampened with normal saline throughout the entire session. The gracilis and semitendinosus tendons were straightened and deposited on the table and then measured in length and width with a millimetric graduated ruler. The orientation of one of them was flipped head-to-tail to uniformize graft diameter before being folded on a high-size PDS suture, corresponding to the in vivo technique for the 4S graft configuration (Figure 1). 4S grafts’ length and diameter were measured.

The two tendons were unfolded and whipstitched together at one end with a resorbable polyfilament 2-0 suture. They were folded in three over a resorbable poly filament size 1 suture before being whipstitched and tubularized at both extremities with a resorbable polyfilament 2-0 suture to constitute the 6S graft (Figure 1). The 2-0 suture was considered small enough so it would not affect significatively the diameter measurement. 6S grafts’ length and diameter were measured.

Lengths and widths of all tendons and grafts were measured with a standard millimetric graduated ruler. Diameters of the 4S and 6S grafts were measured with a *Conmed Sizing Tube set* (Conmed Linvatec, Aurora, Ohio) ranging from 6.5 to 11 mm in increments of 0.5 mm. In order to represent as accurately as possible the in-vivo technique used in our center, tendons and grafts were not pre-tensioned before being measured in length and diameter, other than the usual manual traction needed to pass the graft through the sizing tubes. The strength needed to pull grafts through the tubes was left to the surgeon’s judgment to reflect in vivo reality. As a general principle, when deciding between sizes, surgeons evaluated which graft would optimally fill a tunnel of the size measured, without being unreasonably hard to pull through.

Lengths, widths, and diameters in 4S and 6S configurations were measured independently by three different evaluators (two fellowship-trained sports medicine surgeons and one senior resident), in order to evaluate the inter-observer reliability. Ten 4S grafts were also randomly and blindly re-evaluated in different sessions by each evaluator in order to establish the intra-observer reliability of sizing tube measurements. Blinding was achieved by having the research assistant randomly select the ten grafts to be reassessed, without the observers being aware of which grafts were chosen.

Statistical calculations and analyses were done by a statistician with SPSS (v23.0.0 from IBM). Descriptive statistics were used for the calculation of means, medians, proportions, and confidence intervals. Median values obtained for each sample from the three evaluators were used for diameter calculations, whereas means of the three evaluators’ measures were used for length and width calculations. A Shapiro-Wilk Test was used to assess the normality of the variables. A two-tailed Wilcoxon Signed-Rank Test was used to compare the 4S and 6S graft diameters since both were paired and were considered ordinal variables in the context of this study. A statistical significance threshold of 0.05 was used. Inter and intraobserver reliability of graft diameter measures was evaluated with the help of *Kendall’s coefficient of concordance,* which allows evaluation of the concordance of discrete ordinal measures.

The whole dataset has been published in an open-access data repository [23].

## Results

Thirty-three of the 34 dissected knees were deemed usable for the study. One knee was excluded because of damage caused to the graft while being harvested. Demographic characteristics of the 17 cadavers have been tabulated (Table 1).

**Table 1:** Demographic characteristics.

Number of samples	17 cadavers (33 samples)
Average age, y (average ± *SD*)	76.2 ± 13.6
Body mass index, kg/m^2^ (average ± *SD*)	22.6 ± 4.0
Sex, % men	52.9

Characteristics of the harvested semitendinosus and gracilis tendon are as follow: the semitendinosus tendons had a mean length of 300.9 (*SD* = 31.6) mm and a width of 6.1 (*SD* = 0.9) mm, whereas the gracilis tendons had a mean length of 269.4 (*SD* = 24.4) mm and a width of 4.3 (*SD* = 0.7) mm.

The mean length of 4S grafts is 143.6 mm (*SD* = 15.0) and all of them were over 90 mm long. The mean length of 6S grafts is 96.3 mm (*SD* = 10.3) and 72.7% of them reached the desired length of 90 mm. A minimum semitendinosus length of 253 mm (sensitivity 0.949, specificity 1.000) was required to reliably obtain a 6S graft length of 80, whereas a semitendinosus length of 270.5 mm (sensitivity 0.946, specificity 0.7508) was required to reliably obtain a 6S graft length of at least 90 mm.

Within this population, 69.7% of grafts would not measure more than 8 mm in a 4S configuration, with this proportion dropping to 15.2% in a 6S configuration (Table 2).

**Table 2:** 4S and 6S grafts diameters distribution.

Diameter, mm	No. of 4S (%)	No. of 6S (%)
7.0	9 (27.3%)	0 (0%)
7.5	6 (18.2%)	1 (3.0%)
8.0	8 (24.2%)	4 (12.1%)
8.5	9 (27.3%)	3 (9.1%)
9.0	1 (3.0%)	10 (30.3%)
9.5	0 (0.0%)	8 (24,2%)
10.0	0 (0.0%)	5 (15.2%)
10.5	0 (0.0%)	2 (6.1%)

Legend: Shaded area includes all values exceeding 8 mm.

Medians (range) were used to describe graft diameters since both were paired ordinal variables, and 4S graft diameter data did not follow a normal distribution (Shapiro-Wilk = 0.874, *p* = 0.0012). The median diameter of 4S grafts is 8.0 (range: 7.0–9.0) mm versus 9.0 (range: 7.5–10.5) mm for the 6S grafts, which translates to a 1.5 (range: 0.0–2.0) mm median increase in diameter. The 6S graft configuration significantly increased graft diameter (*p* < 0.001). After being folded in 3, 78.3% of all grafts with a diameter initially considered insufficient reached a size larger than 8 mm. This percentage increases to 92.9% if we consider only those with an initial diameter of 7.5–8.0 mm (Table 3).

**Table 3:** Diameter increase with 6S configuration.

4S Diameter, mm	Detailed 6S Diameters, mm; Frequency, number of grafts		Median increase, mm (range)	Percentage of specimens exceeding 8 mm after 6S configuration		
7	7.5	1	1.5 (0.5–2.0)	55.6%		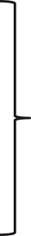 78.3%
	8	3				
	8.5	2				
	9	3				
7.5	8	1	1.5 (0.5–1.5)	83.3%	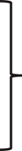 92.9%	
	8.5	1				
	9	4				
8	8.5	0	1.5 (1.0–2.0)	100%		
	9	1				
	9.5	5				
	10	2				
8.5	9	1	1.5 (0.5–2.0)	100%		
	9.5	3				
	10	3				
	10.5	2				
9	9	1	0.0	100%		
Global			1.5 (1.18–1.52)	84.8%		

Legend: Shaded areas include all values exceeding 8 mm.

Inter-rater and intra-rater reliability of diameter measure on 4S grafts, were excellent, with concordances of 0.904 (*p* < 0.001) and 0.917 (*p* < 0.001), respectively.

## Discussion

These results confirm that using a 6S configuration for hamstring grafts consistently and significantly increases the diameter of the graft by a median of 1.5 mm when compared to a 4S configuration. In order to be able to use these results during the per-operative decision-making process, we described the impact of this change in graft configuration using a technique as close as possible to the in-vivo surgical technique.

No other study, to the best of our knowledge, looked at the change in diameter between 4S and 6S configurations. However, our data is consistent with recent studies comparing the average diameter between 4S and 5S grafts. Walczak et al. [21] found a mean increase in diameter of 0.99 mm, whereas Krishna et al. [22] reported a 1.4 ± 0.3 mm in their randomized clinical trial when changing from a 4S to a 5S. In this latter study, the proportion of 5S grafts exceeding 8 mm was 75% while it is 78% in our 6S configuration.

As detailed in the introduction, many studies have shown better biomechanical and clinical results with bigger graft diameters [2–4, 12, 13]. For example, Conte et al. [3] have stated in their 2014 systematic review that grafts of more than 8 mm in diameter are protective against rupture, mainly based on Magnussen et al. [2] and Mariscalco et al. [4] papers. None of the studies comparing functional and clinical outcomes between ACL reconstructions done with 4S and 5S/6S grafts found any difference. Therefore, they concluded that the 5S or 6S configurations remain safe options in case of smaller tendons [24, 25].

Given this information, we wanted to determine the percentage of grafts that reached a diameter of more than 8.0 mm (i.e., minimum 8.5 mm threshold). We found that 78.3% of 4S grafts with a diameter of 8.0 mm or less reached 8.5 mm or more when the 6S configuration was used. This proportion increased to 92.9% if we considered only the 4S grafts of 7.5 or 8 mm, meaning that this 7.5 mm cutoff could be used in an intra-operative decisional algorithm to drive the surgeon into the 6S configuration if a diameter of at least 8.5 mm is desired. It is worth noting that one must be cautious from blindly aiming for a graft of 8.5 mm or more as some knees are smaller and have narrower intercondylar notches. A graft of too large a diameter could lead to impingement and loss of an extension [26]. The prediction of increase in diameter shown in our study could also help to avoid choosing a configuration that could lead to overstuffing of the notch.

The length of tendons also plays a role in this decisional process. The desired graft length changes depending on surgical technique but usually varies between 80 mm [27] and 90 mm for surgeons who use interference screw fixation in the tibia. A cutoff semitendinosus length of 270.5 mm can be used if a 6S graft length of 90 mm is desired and 253 mm for an 80 mm graft length. In scenarios where semitendinosus tendon length would be insufficient, other configuration techniques, like quadricipital tendon supplementation [15], could be used instead of 6S.

### Limitations

The fact that this study is based on cadavers and that the mean age of the donors does not represent the usual ACL tear population can diminish the clinical applicability of its results, mainly when looking at the proportion of initial tendon sizes. The absolute diameter of the tendons used can differ from the one in our aimed population because of age and preservation process. However, since this study measures relative increases in graft diameter values between specific 4S graft diameters and their 6S configuration’s diameter, we believe that the age or sex of the population has a minimal impact on the ability to properly answer the research question.

The total number of samples (*n* = 33) directly limits the numbers per group and might therefore influence the external validity of the experiment. Several questions, such as the complications associated with the technique as well as the real clinical benefit, have not been evaluated and are beyond the scope of this project. As an example, the type of fixation on the femoral side might have an impact on the biomechanical properties of 5S and 6S grafts, as Snow et al. [28] have shown and must be accounted for.

The use of sizing tubes instead of a caliper to measure diameters, as well as the distribution of the data, has forced the use of medians instead of means since the variable was discrete and not normally distributed. The decision to use sizing tubes was taken in order to replicate as closely as possible the in-vivo surgical technique.

Finally, lengths, widths, and diameters were measured in a non-blinded manner and without clearly stated guidelines in regard to the tension needed to pass the graft through the sizing tubes. We tried to minimize the risk of measurement bias by asking two surgeons who specialized in sports surgery and one senior resident to repeat every measure individually, under the supervision of a research assistant. The excellent inter-rater and intra-rater reliability of diameter measurement with sizing tubes of 0.904 (*p* < 0.001) and 0.917 (*p* < 0.001) confirm the minimal effect of this possible bias.

### Conclusion

The 6S hamstring graft configuration increases the graft diameter by a median of 1.5 mm compared to the traditional 4S configuration. It can reliably be used to obtain grafts larger than 8 mm and a length of 90 mm in cases where the semitendinosus measures at least 275 mm and the 4S configuration has a diameter of 7.5 or 8 mm.
